# Supplementary material for: Mycobiomes of two distinct clades of ambrosia gall midges (Diptera: Cecidomyiidae) are species-specific in larvae but similar in nutritive mycelia
Source: Microbiol Spectr. 2023 Dec 14;12(1):e02830-23. doi: 10.1128/spectrum.02830-23 (PMC10782975; doi:10.1128/spectrum.02830-23)

**Supplementary Information for**

Mycobiomes of two distinct clades of ambrosia gall midges (Diptera: Cecidomyiidae) are species-specific in larvae but similar in nutritive mycelia

Authors: Petr PYSZKO, Hana ŠIGUTOVÁ, Miroslav KOLAŘÍK, Martin KOSTOVČÍK, Jan ŠEVČÍK, Martin ŠIGUT, Denisa VIŠŇOVSKÁ, & Pavel DROZD

Corresponding author: Petr Pyszko

Email: petr.pyszko@osu.cz

**This file includes Supplementary Material 2A.** Taxonomic identity of species significantly indicative for gall surfaces, gall interiors and AGM larvae

**Supplementary material 2A**. Taxonomic identity of ITS2 sequences ascribable to the *Botryosphaeria* genus.

There were five ASVs of ITS2 sequences that were similar to the *Botryosphaeria* species. The only variable positions were found at the beginning and end of the read sequence (Figure S1). They were compared with the best hits from the NCBI GenBank representing the type and other reference strains (Table S1). Sequences were aligned in MAFFT 6 using the G-INS-i strategy (Katoh and Standley 2013). Maximum likelihood (ML) phylogenetic analyses were performed using the PhyML 3.1. (Guindon *et al.* 2010), using 500 bootstrap replicates. The tree was rooted in *Botryosphaeria mamane.* The ITS2 sequences from our study were identical to the type strain of *B. dothidea* (AY259092) and conspecific isolates from gall midges (Adair *et al.* 2009; Kobune *et al.* 2012; Lebel, Peele and Veenstra 2012; Bernardo *et al.* 2018; Zimowska *et al.* 2020) (Figure S2), and also to related species such as *B. auasmontanum*, *B. fabicerciana*, *B. scharifii*, *B. ramosa* and *B. fusispora*. None of these species have been reported as AGM symbionts and are known only from outside Europe (Phillips *et al.* 2013). Therefore, we assume that the dominant AGM symbiont in our study is *B. dothidea*.

**Table S1.** List of sequences compared with *Botryosphaeria* ITS2 sequence reads.

| **Sequence accession** | **Species name** | **Strain** | **Note** | **Country** | **Reference** |
| --- | --- | --- | --- | --- | --- |
| KF766167 | *B. auasmontanum* | CMW 5413 | ex-type strain | Namibia | (Slippers *et al.* 2013) |
| AY236949 | *B. dothidea* | CMW 8000 (CBS 115476) | ex-type strain | Switzerland | (Slippers *et al.* 2004) |
| AY259092 | *B. dothidea* | CBS 110302 | reference strain | Portugal | (Phillips *et al.* 2013) |
| HQ332197 | *B. fabicerciana* | CBS 127193 | ex-type strain | China | (Chen *et al.* 2011) |
| JQ772020 | *B. scharifii* | CBS 124703 (IRAN 1529C) | ex-type strain | Iran | (Abdollahzadeh, Zare and Phillips 2013) |
| EU144055 | *B. ramosa* | CBS 122069 ex-type | ex-type strain | Australia | (Phillips *et al.* 2013) |
| JX646789 | *B. fusispora* | MFLUCC 10-0098 ex-type | ex-type strain | Thailand | (Phillips *et al.* 2013) |
| EU520152 | *B. dothidea* | NW276 |  | China | unpublished |
| MN634017 | *B. dothidea* | IRNKB213 |  | Iran | (Sohrabi *et al.* 2020) |
| KC960907 | *B. dothidea* | J531 |  | China | (Zhai *et al.* 2014) |
| AB645749 | *B. dothidea* | FFPRI411072 | ambrosia gall | Japan | (Kobune *et al.* 2012) |
| MN731278 | *B. dothidea* | Thgl/10 | ambrosia gall | Italy | (Zimowska *et al.* 2020) |
| MF092879 | *B. dothidea* | gall25 | ambrosia gall | Italy | (Bernardo *et al.* 2018) |
| KY368175 | *B. dothidea* | Th.L 12.509 | ambrosia gall | Poland | (Zimowska *et al.* 2017) |
| KT823763 | *B. dothidea* | Gall 5 | ambrosia gall | Switzerland | (Meyer, Gallien and Prospero 2015) |
| JX401387 | *Uncultured Botryosphaeria clone* | 21mtl2101 | ambrosia gall larva | Australia | (Lebel, Peele and Veenstra 2012) |
| MK178548 | *B. dothidea* | LTNM6 | ambrosia gall | USA | (Park *et al.* 2019) |
| KU377487 | *B. mamane* | D007 | ambrosia gall | Venezuela | (Castillo *et al.* 2016) |

**Figure S1.** Nucleotide variability in ASVs showing similarity to *Botryosphaeria* species.


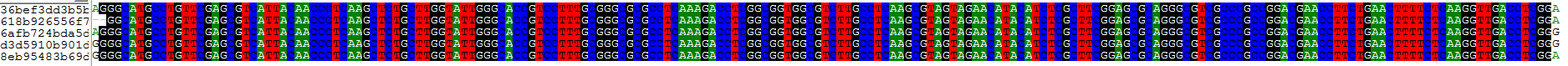


**Figure S2.** Phylogenetic tree showing relatedness of ITS2 sequence reads from this study and representative *Botryosphaeria* sequences from NCBI Genbank.


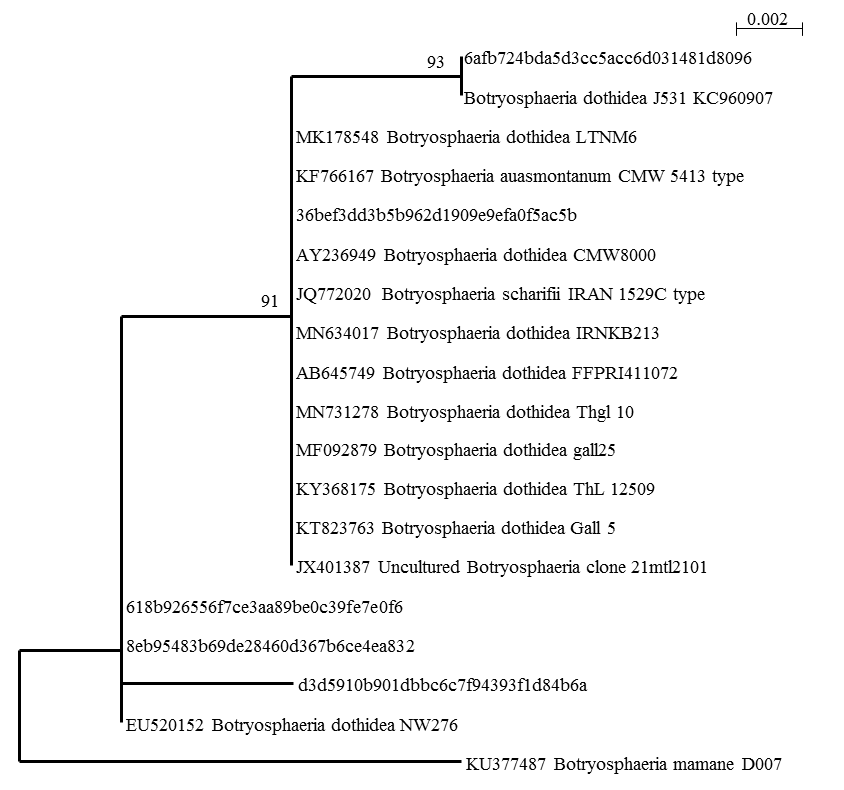

Supplement: Supplemental Material S2A — Taxonomic identity of ITS2 sequences ascribable to the Botryosphaeria genus. [file spectrum.02830-23-s0001.docx]
