# Supplementary material for: Mycobiomes of two distinct clades of ambrosia gall midges (Diptera: Cecidomyiidae) are species-specific in larvae but similar in nutritive mycelia
Source: Microbiol Spectr. 2023 Dec 14;12(1):e02830-23. doi: 10.1128/spectrum.02830-23 (PMC10782975; doi:10.1128/spectrum.02830-23)
Supplement: Supplemental Material S2C — Taxonomic identity of cultures isolated from gall interior and larvae. [file spectrum.02830-23-s0002.docx]

**Supplementary Information for**

Mycobiomes of two distinct clades of ambrosia gall midges (Diptera: Cecidomyiidae) are species-specific in larvae but similar in nutritive mycelia

Authors: Petr PYSZKO, Hana ŠIGUTOVÁ, Miroslav KOLAŘÍK, Martin KOSTOVČÍK, Jan ŠEVČÍK, Martin ŠIGUT, Denisa VIŠŇOVSKÁ, & Pavel DROZD

Corresponding author: Petr Pyszko

Email: petr.pyszko@osu.cz

**This file includes Supplementary Material 2C:** Taxonomic identity of cultures isolated from gall interior and larvae.

**Supplementary material 2C**. Taxonomic identity of cultures isolated from gall interior and larvae.

^1^ For the best hit, the species name, strain, sequence accession number and sequence similarity (%) is shown.

| **Strain no.** | **Host insect** | **Tissue type** | **Genbank Accession no.** | **Genbank best hit^1^** | **Species identity** |
| --- | --- | --- | --- | --- | --- |
| FABA-FM08 | *Asphondylia miki* | Gall interior | OR797332 | ITS: *Akanthomyces muscarius*, IMI 068689 (TYPE material) 99.4NR_111096.1 | *Akanthomyces cf. muscarius* |
| A.e.H1, A.e.H2 | *Asphondylia echii* | Gall interior | To be added | TEF1a: *Alternaria* *alternata,* ICMP 2015, OM522544, 100%  ITS: *Alternaria* *alternata,* ISHAM-ITS_ID MITS64, KC253941, 100% | *Alternaria alternata* |
| CARO-FM02 | *Lasioptera carophila* | Gall interior | OR797337 | ITS: *Alternaria* *destruens*, ATCC 204363 (TYPE material), NR_137143 100.00%  *Alternaria alternata*, CBS 126910, MH864334, 100% | *Alternaria alternata* species complex |
| ECHI-FM02 | *Asphondylia echii* | Gall interior | OR797343 | ITS: *Alternaria* *destruens*, ATCC 204363 (TYPE material), NR_137143 100.00%  *Alternaria alternata*, CBS 126910, MH864334, 100% | *Alternaria alternata species complex* |
| CARO-FM06_13 | *Lasioptera carophila* | Gall interior | OR797336 | ITS: *Alternaria* *rosae* CBS 121341 (TYPE material), NR_136017.1, 100% | *Alternaria rosae* |
| A.e.L1 | *Asphondylia echii* | larva | OR797316 | TEF1a: *Alternaria* *arbusti* CBS 596.93, FJ214924, 97.2%  ITS: identical to several species, e.g. *A*. rosiae CBS 121341 (type) | *Alternaria* sp. |
| CARO-FM04 | *Lasioptera carophila* | Gall interior | OR797325 | ITS: *Aureobasidium* *subglaciale* CBS 123387 (TYPE material), NR_147323.1, 99.78% | *Aureobasidium subglaciale* |
| FABA-FM04 | *Asphondylia miki* | Gall interior | OR797331 | ITS: *Aureobasidium* *subglaciale* CBS 123387 (TYPE material), NR_147323.1, 99.78% | *Aureobasidium subglaciale* |
| L.r.L1 | *Lasioptera rubi* | larva | OR797320 | ITS: *Botryosphaeria* *dothidea,* CBS 110484, MT587330, 100%  TEF1a: *Botryosphaeria* *dothidea,* GZHGS-2017-010, MK108374, 99.8% | *Botryosphaeria dothidea* |
| LA029 | *Lasioptera artemisiae* | gall | Identical with OR797320 | ITS: *Botryosphaeria* *dothidea*, CBS 110484, MT587330, 100%  TEF1a: *Botryosphaeria* *dothidea*, GZHGS-2017-010, MK108374, 99.8% | *Botryosphaeria dothidea* |
| ECHI-FM01 | *Asphondylia echii* | Gall interior | OR797338 | ITS: *Cladosporium* *asperulatum*, CPC 14040 (TYPE material), NR_119836, 100%  *Cladosporium* *globisporum*, CBS 812.96 (TYPE material), NR_111534.1, 100% | *Cladosporium cladosporoides* species complex |
| VERB-FM01 | *Asphondylia verbasci* | Gall interior | OR797340 | ITS: *Cladosporium* *asperulatum*, CPC 14040 (TYPE material), NR_119836, 100%  *Cladosporium* *globisporum*, CBS 812.96 (TYPE material), NR_111534.1, 100% | *Cladosporium cladosporoides* species complex |
| CARO-FM01 | *Lasioptera carophila* | Gall interior | OR797339 | ITS: *Cladosporium* *asperulatum*, CPC 14040 (TYPE material), NR_119836, 100%  *Cladosporium* *globisporum*, CBS 812.96 (TYPE material), NR_111534.1, 100% | *Cladosporium cladosporoides* species complex |
| ERYN-FM01 | *Lasioptera eryngii* | Gall interior | OR797341 | ITS: *Cladosporium* *asperulatum*, CPC 14040 (TYPE material), NR_119836, 100%  *Cladosporium* *globisporum*, CBS 812.96 (TYPE material), NR_111534.1, 100% | *Cladosporium cladosporoides species complex* |
| FABA-FM01 | *Asphondylia miki* | Gall interior | OR797342 | ITS: *Cladosporium* *asperulatum*, CPC 14040 (TYPE material), NR_119836, 100%  *Cladosporium* *globisporum*, CBS 812.96 (TYPE material), NR_111534.1, 100% | *Cladosporium cladosporoides species complex* |
| PHRAGM-FM04 | *Lasioptera arundinis* | Gall interior | OR797324 | ITS: *Occultifur* *kilbournensis* NRRL Y-63695, NR_155564.1 (TYPE material), 85.68% | *Cystobasidiales* sp. |
| ECHI-FM04 | *Asphondylia echii* | Gall interior | OR797326 | ITS: *Filobasidium* *magnum*, CBS 140 ITS region; from (TYPE material), NR_130655, 99.81% | *Filobasidium magnum* |
| L.a.H2 | *Lasioptera artemisiae* | larva | OR797321 | TEF1a: *Fusarium* *arthrosporioides* BBA 63782, KC999487, 100%)  ITS: identical with various species, e.g.  *F. acuminatum* OP897709, (100%) | *Fusarium arthrosporioides* |
| L.a.H1 | *Lasioptera artemisiae* | larva | OR797318 | TEF1a: *Fusarium* *avenaceum* NRRL 54939, MH582391, 99.8%  ITS: identical with various species, e.g. *Fusarium* *tricinctum* WZ-216, MN856343 | *Fusarium avenaceum* |
| L.a.H3 | *Lasioptera artemisiae* |  | OR797322 | TEF1a: *F. avenaceum*, ICMP:1789, [MG857132.1](https://www.ncbi.nlm.nih.gov/nucleotide/MG857132.1?report=genbank&log$=nuclalign&blast_rank=8&RID=ZCMA3FXE013),99.8%  ITS: *F*. *avenaceum*, NRRL36374, OL832183, 100% | *Fusarium avenaceum* |
| A.e.L2, | *Asphondylia echii* | larva | OR797317 | TEF1a: *Fusarium* *juglandicola* UBOCC-A-102014, MZ078245, 100%  ITS: *Fusarium* *aconidiale* CPC:37959, MZ064464, 100% | *Fusarium juglandicola* |
| L.r.L3 | *Lasioptera rubi* | larva | Identical with A.e.L2 | TEF1a: *Fusarium* *juglandicola* UBOCC-A-102014, MZ078245, 100%  ITS: *Fusarium* *aconidiale* CPC:37959, MZ064464, 100% | *Fusarium juglandicola* |
| VERB-FM06 | *Asphondylia verbasci* | Gall interior | OR797333 | ITS: *Heterophoma* *sylvatica*, CBS 874.97, NR_136006.1 (TYPE material), 100.00% | *Heterophoma sylvatica* |
| GS30-M09, GS30-M09 | *Lasioptera arundinis* | Gall interior | OR797334 | ITS: *Mucor* *circinelloides*, CBS 195.71, MH860064, 99.81% | *Mucor circinelloides* |
| PHRAGM-FM09 | *Lasioptera arundinis* | Gall interior | OR797335 | ITS: *Mucor fragilis* IA1I1F3, KX421452.1, 100.00% | *Mucor fragilis* |
| VERB-FM04 | *Asphondylia verbasci* | Gall interior | OR797323 | ITS: *Papiliotrema flavescens*, CBS 942 (TYPE material), NR_130696, 99.34% | *Papiliotrema flavescens* |
| CARO-FM03 | *Lasioptera carophila* | Gall interior | OR797329 | ITS: *Rhodosporidiobolus azoricus*, CBS 8949 (TYPE material), NR_155729.1, 100.00% | *Rhodosporidiobolus azoricus* |
| ECHI-FM03 | *Asphondylia echii* | Gall interior | OR797330 | ITS: *Rhodosporidiobolus azoricus*, CBS 8949 (TYPE material), NR_155729, 99.6% | *Rhodosporidiobolus azoricus* |
| VERB-FM03 | *Asphondylia verbasci* | Gall interior | OR797328 | ITS: *Rhodosporidium babjevae*, CBS 7808 (TYPE material), NR_077096.1, 99.44% | *Rhodosporidium babjevae* |
| L.a.H4 | *Lasioptera artemisiae* | larva | OR797319 | ITS: *Sarocladium* *bacillisporum,* CBS 425.67, MH859020, 100%  TEF1a: Cadophora luteo-olivacea CBS:141.41, JN808856, 90% | *Sarocladium bacillisporum* |
| PHRAGM_-FM08 | *Lasioptera arundinis* | Gall interior | OR797327 | ITS: *Sarocladium* *spinificis*, KF269096.1 100.00% | *Sarocladium spinificis* |
