# Supplementary material for: Mycobiomes of two distinct clades of ambrosia gall midges (Diptera: Cecidomyiidae) are species-specific in larvae but similar in nutritive mycelia
Source: Microbiol Spectr. 2023 Dec 14;12(1):e02830-23. doi: 10.1128/spectrum.02830-23 (PMC10782975; doi:10.1128/spectrum.02830-23)

**Supplementary Information for**

Mycobiomes of two distinct clades of ambrosia gall midges (Diptera: Cecidomyiidae) are species-specific in larvae but similar in nutritive mycelia

Authors: Petr PYSZKO, Hana ŠIGUTOVÁ, Miroslav KOLAŘÍK, Martin KOSTOVČÍK, Jan ŠEVČÍK, Martin ŠIGUT, Denisa VIŠŇOVSKÁ, & Pavel DROZD

Corresponding author: Petr Pyszko

Email: petr.pyszko@osu.cz

**This file includes Supplementary Material 1** (Figures S1 to S4)

**Fig S1.** Rarefaction curves performed at the amplicon sequence variant (ASV) level for samples from individual gall compartments showing the sufficient sequencing depth


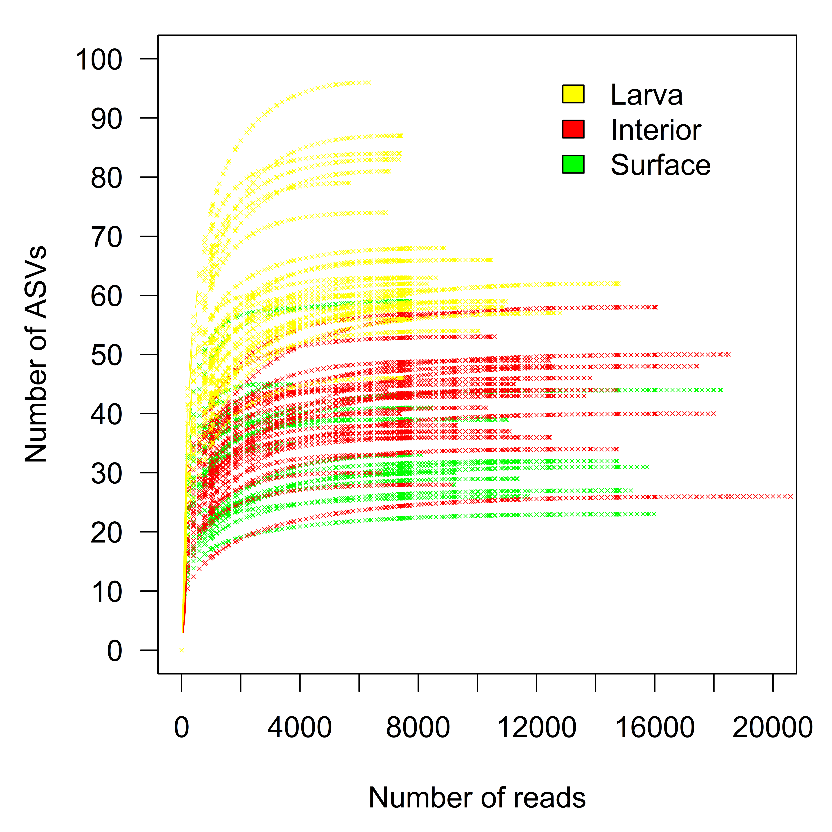


**Fig. S2.** The gall compartment (i.e., gall surface, interior and larva) was the best explanatory variable of mycobiome composition, explaining 13.45% of variability (df = 90, F = 9.24, *P* = 0.001), followed by gall species, explaining 14.94% of variability (df = 85, F = 4.10, *P* = 0.001), and their interaction (17.00% of variability, df = 75, F = 2.33, *P* = 0.001). Due to the significant interaction, we analyzed each gall compartment separately, and the greatest difference among individual species was detected at the level of larvae (AGM species explaining 43.31% of variability, df = 25, F = 3.82, *P* = 0.001), followed by gall surface (i.e. different host plants, 37.36% of variability, df = 25, F = 2.98, *P* = 0.001), while the lowest but still significant differences among AGM species were in gall interiors (29.38% of variability, df = 25, F = 3.82, *P* = 0.001). Principal coordinates analysis plots based on partial-canonical correspondence analysis show dissimilarity in the composition of fungi among mycobiomes of gall surface, interior and gall midge larvae at the level of a) ASVs (F = 3.60, P = 0.001), and b) species with binarized data (F = 3.20, P = 0.001)


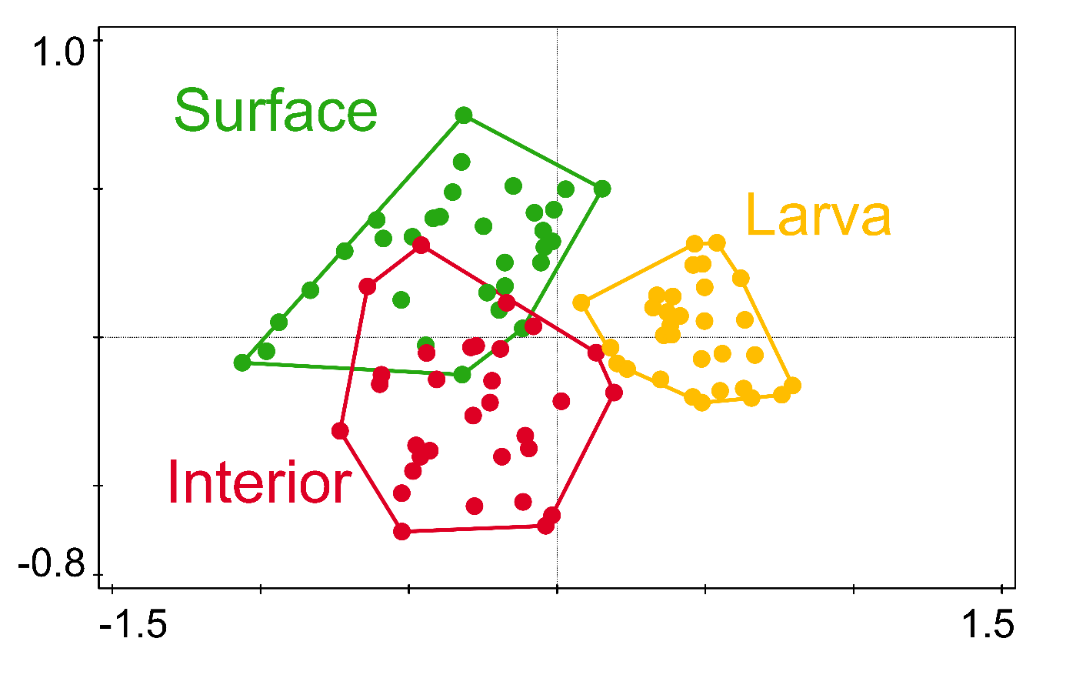

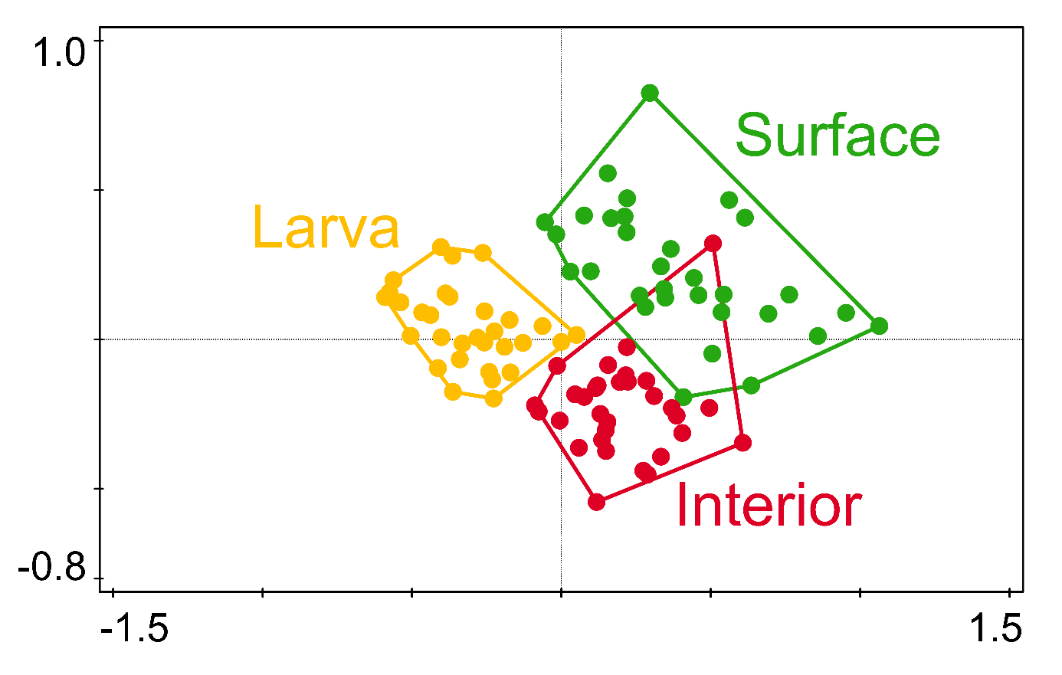


b)

a)

**Fig. S3.** Species accumulation curves for gall mycobiomes of individual gall parts for a) *Asphondylia echii*, b) *A. miki*, c) *A. verbasci*, d) *Lasioptera arundinis*, e) *L. carophila*, f) *L. eryngii*


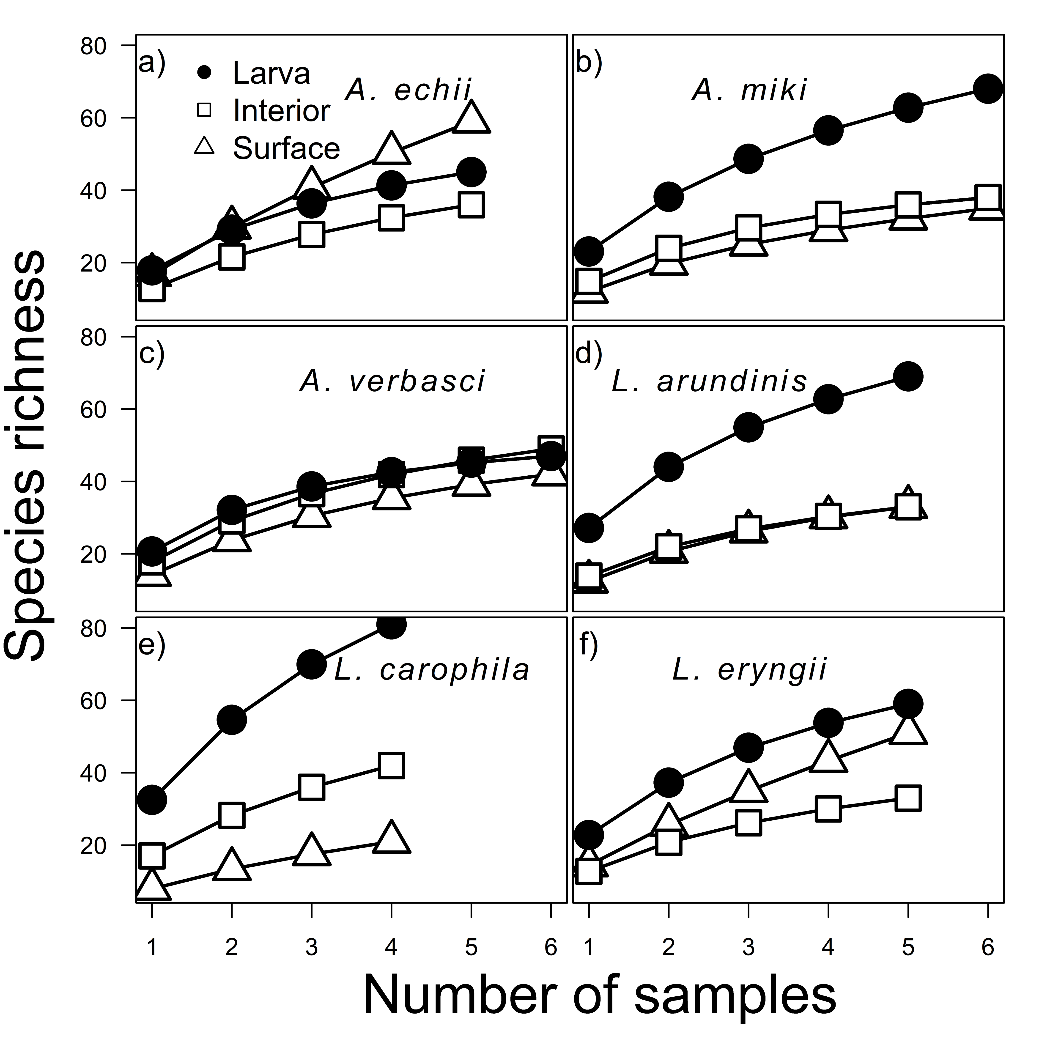


**Fig. S4.** Rarefied fungal ASVs richness for gall mycobiomes of individual gall parts for individual AGM species: a) *Asphondylia echii*, b) *A. miki*, c) *A. verbasci*, d) *Lasioptera arundinis*, e) *L. carophila*, f) *L. eryngii*. The gall part was the only significant explanatory variable of fungal richness, explaining 51.91% of the variability (df = 90, F = 42.24, *P* < 0.001). The highest richness was associated with larvae. In comparison to the surface, the richness of the gall interiors did not differ (df = 90, t = -0.05, *P* = 0.962), whereas larval richness differed significantly (df = 90, t = 7.96, *P* < 0.001). Significant differences are indicated by different letters


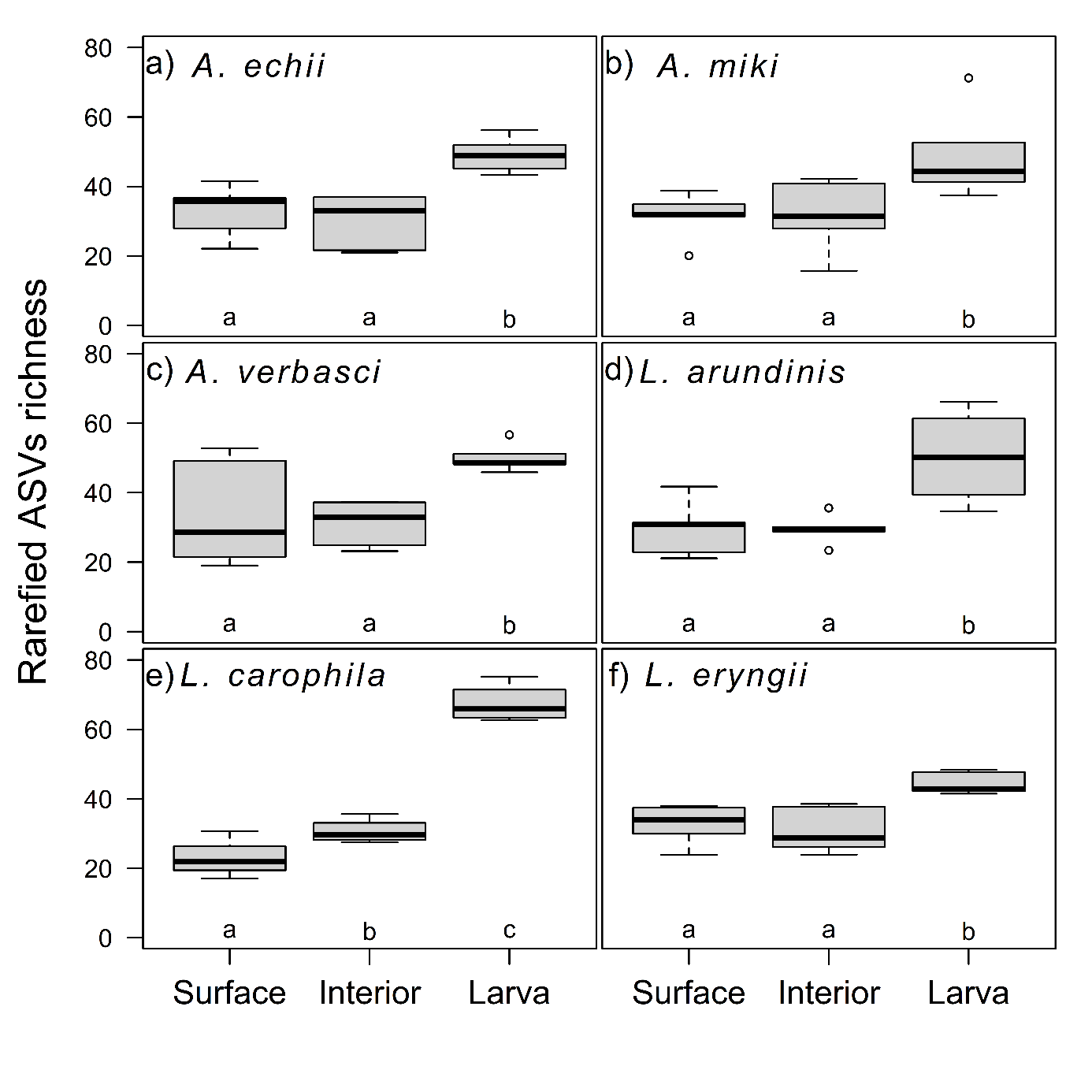

Supplement: Supplemental Material S1 — Figures S1 to S4. [file spectrum.02830-23-s0003.docx]
