# Supplementary material for: Mycobiomes of two distinct clades of ambrosia gall midges (Diptera: Cecidomyiidae) are species-specific in larvae but similar in nutritive mycelia
Source: Microbiol Spectr. 2023 Dec 14;12(1):e02830-23. doi: 10.1128/spectrum.02830-23 (PMC10782975; doi:10.1128/spectrum.02830-23)
Supplement: Supplemental Material S2B — Taxonomic identity of species significantly indicative for gall surfaces, gall interiors, and AGM larvae. [file spectrum.02830-23-s0004.docx]

**Supplementary Information for**

Mycobiomes of two distinct clades of ambrosia gall midges (Diptera: Cecidomyiidae) are species-specific in larvae but similar in nutritive mycelia

Authors: Petr PYSZKO, Hana ŠIGUTOVÁ, Miroslav KOLAŘÍK, Martin KOSTOVČÍK, Jan ŠEVČÍK, Martin ŠIGUT, Denisa VIŠŇOVSKÁ, & Pavel DROZD

Corresponding author: Petr Pyszko

Email: petr.pyszko@osu.cz

**This file includes Supplementary Material 2B.** Taxonomic identity of species significantly indicative for gall surfaces, gall interiors and AGM larvae

**Supplementary material 2B**. Taxonomic identity of species significantly indicative for Gall surfaces, Gall interiors and AGM larvae of *Asphondylia echii*, *Asphondylia miki*, *Asphondylia verbasci*, *Lasioptera arundinis*, *Lasioptera carophila* and *Lasioptera eryngii*. It presents the primary data for Table 1. The identity of *Botryosphaeria* is discussed in Supplementary material 1. The best hits (Genbank, UNITE) originating from the type strains or other well documented material from taxonomic studies were selected for presentation.

| **Sequence ID** | **Indicative for – substrate type** | **Indicative for – AGM** **species** | **The best blast hit** | **Taxonomic identity** |
| --- | --- | --- | --- | --- |
| 90d76d3094f07b3c49716abfcfb8492b | Gall surfaces (*A. echii*)  AGM larvae | *A. echii, A. miki, L. arundinis, L. carophila, L. eryngii* | 100%, *Alternaria alternata* CBS 126910; *Alternaria angustiovoidea* CBS 195.86 (type), MH861939; 100%, *Alternaria destruens* ATCC 204363 (type), NR_137143 | *Alternaria sp.* (*A. alternatra* species complex) |
| 1fcd05d0b4c69c39e65d5afded0da074 | AGM larvae | *A. echii, A. miki, A. verbasci, L. eryngii, L. arundinis* | 100%, *Filobasidium oeirensis* XJ10A2, HE650884 | *Filobasidium oeirense* |
| 5d3e1864ef52b7bbd7d141f0b95a163e | AGM larvae | *A. echii, A. miki, L. eryngii, L. carophila, L. arundinis* | 100%, *Fusarium sporotrichioides* CBS 180.32, MH855269 | *Fusarium sporotrichioides* |
| 0eb0f9055b30f29822eac6372ced3f14 | AGM larvae | *A. echii* | 100%, *F. avenaceum* CBS 128538, MH864972; 100% *F. tricinctum* CBS 261.51, MH856847; 100% *F. lateritium* CBS 122446 | *Fusarium* sp. (*F. avenaceum* species complex) |
| 43925bad576e342736e3e1b5223b7639 | AGM larvae | *A. miki, L. arundinis, L. eryngii* | 99%, *Alternaria indefessa* CBS 536.83, MH861641; 99%, *Alternaria botryospora* CBS 478.90, MH862228 | *Alternaria* sp. |
| 47cc5099628737a55d3fa82891b024c6 | AGM larvae | *A. miki* | 100% *Periconia byssoides* CBS 685.70, MH859902 | *Periconia byssoides* |
| 979f9402723408c308f8a9f9512a2076 | AGM larvae | *A. miki, L. arundinis, L. carophila* | 100%, *Tilletiopsis washingtonensis* AFTOL-ID 868, DQ835994 | *Tilletiopsis washingtonensis* |
| cfe812b84204fea0c9c3250cae16adaa | AGM larvae | *A. miki* | 99%, *Neosetophoma xingrensis* GZCC 18-0110 (type), MH018135 | *Neosetophoma* sp. |
| 979f9402723408c308f8a9f9512a2076 | Gall surfaces | *A. verbasci* | 94%, *Lasiodiplodia parva* CBS 456.78, MH861166 | Botryosphaeriaceae sp. |
| 5a9fbbed7556f8abcc8ff49786274edd | Gall surfaces | *A. verbasci* | 99.7%, *Cladosporium antarcticum* CBS 690.92 (type), NR_121332 | *Cladosporium* sp. 1 |
| 9eb4329cca14c07f665a91a16ce33d7c | Gall surfaces  AGM larvae | *A. verbasci*  *L. eryngii* | 100%, *Cladosporium subuliforme* CBS 126500, MH864124; 100% *Cladosporium asperulatum* CBS 126340, MH863916 | *Cladosporium sp.* 2 |
| fb0dcf9010bb8ea5de82c5ccf22547cf | AGM larvae | *A. verbasci, L. eryngii* | 100%, *Filobasidium wieringae* CBS 1937 (type), NR_077105.1 | *Filobasidium wieringae* |
| 47e640c364f12d4913fbef2ae2742385 | AGM larvae | *A. verbasci* | 100%, *Ramularia abscondita* CBS:114727 (type), NR_154942  100%, *Ramularia calcea* CBS:101612 KJ504785.1; 100% *Ramularia lactea* CBS:114442, KP894229 | *Ramularia* sp., |
| eb5eb0c8fbb80e2124cf32e45ac986af  a9464cb74885acf48b0a664ad53e183a | AGM larvae | *A. verbasci* | 100%, *Rhodotorula glutinis* CBS 20 (type), NR_073294; 99.7% *Rhodotorula graminis* CBS 2826 (type), NR_073273 | *Rhodotorula glutinis* |
| 2cbbe38ad49d8c4bad62ca7a1cf3fff8 | AGM larvae | *A. verbasci, L. arundinis* | 100%, *Heterophoma verbascicola* CGMCC 3.18364 (type); 100%, *Heterophoma* sylvatica CBS 874.97, NR_136006 | Heterophoma sp. |
| ff6c3a0a4c066e2fc01857c062ee4a10 | Gall interior | *A. verbasci, L. carophila* | 97.58% *Myrmecridium phragmiticola* CPC 36367 (type), NR_170826.1 | *Myrmecridium* sp. |
| 752a7c30e2f4695bd52afe56bcd10b55 | Gall interior | *Lasioptera arundinis* | 100%, *Cercospora zebrina* CBS 113070, JX143745 | *Cercospora zebrina* |
| 26d6fd67c95a1c6e09a748989a8b7994 | AGM larvae | *L. arundinis* | 100%, *Mucor fragilis*, FFI 5, AJ608958 | *Mucor fragilis,* |
| f7cb27972748505b069e674bde327f5a | AGM larvae | *L. arundinis* | 95%, *Apenidiella foetida* FMR 17266 (type), NR_165516 | *Apenidiella* sp. 1 |
| d66047916d650bc5ba9a43db7d5fb591 | AGM larvae | *L. arundinis* | 100%, *Pseudopithomyces rosae* MFLU 18-0109 (type), NR_157539 | *Pseudopithomyces rosae* |
| 9d766367e5b044654f91e9fbcec91207 | AGM larvae | *L. arundinis, L. eryngii* | 100%, *Aureobasidium pullulans* CBS 584.75 (type), NR_144909 | *Aureobasidium pullulans* |
| 8bfc9ed53202c0c79bc934549eedc097 | AGM larvae | *L. arundinis* | 96%, *Apenidiella strumelloidea*, CBS 114484, NR_145090 | *Apenidiella* sp. 2 |
| a53c581eb19c2c9bb0b834ae520bb76d | AGM larvae | *L. carophila* | 100%, *Botrytis cinerea* CBS 261.71, MH860108 | *Botrytis cinerea* |
| 15c854418ddb94bf2960085e99d896c6 | AGM larvae | *L. carophila* | 97.3%, *Papiliotrema frias* CRUB 1250, GU997162 | *Papiliotrema frias* |
|  | Gall interior | *L. arundinis* | 100%, *Cercospora beticola* CBS 116456 (type), NR_121315; 100%, *C.* *kikuchii* strain Y. H. Yeh I0916, MK336504; 100%, NR_147293.1 *C. glycinicola* CPC 23912 (type), NR_147293 | *Cercospora* sp. (*C*. *beticola* specis complex) |
| 4f16e7f9672040390711d187e9d6f0a5 | AGM larvae | *L. eryngii* | 99.49%, *Periconia byssoides* CSB F011*,* KU574712 | *Periconia* sp. |
| 763c141141176695f0502693df17f3ed | AGM larvae | *L. eryngii, L. arundinis* | 100%, *Pseudopithomyces rosae* MFLU 18-0109 (type), NR_157539 | *Pseudopithomyces rosae* |
| f5013f87e3c86c1b63158c78dc39e400 | AGM larvae | *L. eryngii* | 99.5%, *Septoria pseudonapelli* CBS 128664 (type), NR_156566; 99%, *Apseudocercosporella trigonotidis* CPC 10865, KX287276 | unkn. Mycosphaerellaceae |

>1643ad5df7184fb034a5357c99ad00b9

CTTGGTATTCCGAGGGGCATGCCTGTTCGAGCGTCATTTCACCACTCAAGCCTCGCTTGGTATTGGGCGCCGCGGTGTTCCGCGCGCCTCAAAGTCTCCGGCTGAGCTGTCCGTCTCTAAGCGTTGTGATTTCATTAATCGCTTCGGAGCGCGGGCGGTCGCGGCCGTTAAATCTTTCACAAGGTTGACCTCGGATCAGGTAGGGATA

>979f9402723408c308f8a9f9512a2076

ATTCTCCACCCCCATGTCTTTTGTCACCAAAGGACGTGCTTGGGGCGGGTCCATGGGCATCTTTGCCAGTCTTCGGACAGGCTTGCCTTAAAAGTATTAGCTGGACTGCATCTGCGATGCATTGGTTCTACTCAACGTGATAAGTTCATTCGTTGAGGACGGCTTCACGGCCGGCCAAGCTACAATGCGATCAATAGTCTGCTTCTAACCCGGCGCGGAGTGCCTGCACTCCAACCCAACTTAACG

>4f16e7f9672040390711d187e9d6f0a5 GTGGGGCATGCCTGTTCGAGCGTCATTTACACCCTCAAGCTCTGCTTGGTGTTGGGCGTCTGTCCCGCCTCCGTGCGCGGACTCGCCTCAAAGTCATTGGCAGCGGTCTCGTCGGCTTCTCGCGCAGCACATTTGCGCTTCTTGGAGCCCCGGCGGATCAGCGTCCAGCAAGCAATTTCATGACTTTACCTCGGATC

>9d766367e5b044654f91e9fbcec91207

GAGGGGCATGCCTGTTCGAGCGTCATTACACCACTCAAGCTATGCTTGGTATTGGGCGTCGTCCTTAGTTGGGCGCGCCTTAAAGACCTCGGCGAGGCCACTCCGGCTTTAGGCGTAGTAGAATTTATTCGAACGTCTGTCAAAGGAGAGGAACTCTGCCGACTGAAGCCTTTATTTTTCTAGGTTGACCTCGGATC

>d66047916d650bc5ba9a43db7d5fb591

CGTGGGGCATGCCTGTTCGAGCGTCATCTACACCCTCAAGCTCTGCTTGGTGTTGGGCGTCTGTCCCGCCTCCGCGCGTGGACTCGCCCCAAATTCATTGGCAGCGGTCCTTGCCTCCTCTCGCGCAGCACATTGCGCTTCTTGAGGGGCTCCGGTTCGCGTCCAACAAGCACATTTACCGTCTTTGACCTCGGATCA

>752a7c30e2f4695bd52afe56bcd10b55

CTTGGTATTCCGAGGGGCATGCCTGTTCGAGCGTCATTTCACCACTCAAGCCTCGCTTGGTATTGGGCGCCGCGGTGTTCCGCGCGCCTCAAAGTCTCCGGCTGAGCTGTCCGTCTCTAAGCGTTGTGATTTCATTAATCGCTTCGGAGCGCGGGCCGTCGCGGCCGTTAAATCTTTCACAAGGTTGACCTCGGATCAGGTAGGGATA

>f5013f87e3c86c1b63158c78dc39e400 TTGGTATTCCGAAGGGCATGCCTGTTCGAGCGTCATTTCACCACTCAAGCCTGGCTTGGTATTGGGCGCCGCGGTCGATCCACGCGCCTCAAAGTCTCCGGCTGAGCTGTCCGTCTCCAAGCGTTGTGATTTCATTAATCGCTTCGGAGTGCGGGCGGCCGCGGCCGTTAAATCTTTCACAAGGTTGACCTCGGATCAGGTAGGGAT

>763c141141176695f0502693df17f3ed

CCGTGGGGCATGCCTGTTCGAGCGTCATCTACACCCTCAAGCTCTGCTTGGTGTTGGGCGTCTGTCCCGCCTCCGCGCGTGGACTCGCCCCAAATTCATTGGCAGCGGTCCTTGCCTCCTCTCGCGCAGCACATTGCGCTTCTTGAGGGGCTCCGGTTCGCGTCCAACAAGCACATTTACCGTCTTTGACCTCGGATCAG

>eae6286aa7e099a461da3a8d062dbc57

GTGGGGCATGCCTGTTCGAGCGTCATTTACACCCTCAAGCTCTGCTTGGTGTTGGGCGTCTGTCCCGCCTCCGTGCGCGGACTCGCCTCAAAGTCATTGGCAGCGGTCTCGTCGGCTTCTCGCGCAGCACATTTGCGCTTCTTGGAGCCCCGGCGGATCAGCGTCCAGCAAGCAATTTCATGACTTGACCTCGGATC

>15c854418ddb94bf2960085e99d896c6

AATCTCAATCCCCCCGGGTTTTCTGAACCCGAGGTGGACTTGGACATGGGTGTCTGCCGTCTTGTACGGCTCGCCTTAAATGACTCAGTGGGATCTTCAGCATCCGTGGCAGACGTAATAAGTTTCGTCTCGTCCCTTGCTGTGAGGACCGCTCATAACCTGCCATCGCGCACCACTTTTGA

>a53c581eb19c2c9bb0b834ae520bb76d

TTGGTATTCCGGGGGGCATGCCTGTTCGAGCGTCATTTCAACCCTCAAGCTTAGCTTGGTATTGAGTCTATGTCAGTAATGGCAGGCTCTAAAATCAGTGGCGGCGCCGCTGGGTCCTGAACGTAGTAATATCTCTCGTTACAGGTTCTCGGTGTGCTTCTGCCAAAACCCAAATTTTTCTATGGTTGACCTCGGATCAGGTAGGGAT

>8bfc9ed53202c0c79bc934549eedc097

TATTCCGGGGGGCATGCCTGTTCGAGCGTCATTACACCAATCAAGCCCCGGCTTGGTCTTGGGCGTCGCGGTCCGCCGCGTGCCTCAATGTCGCCGGCTGGTGCGTCCGTCTCCGAGCGTCGTGACTTCATTGTTCGCTTCTGGAGGCCGGACGGGCCACGCCGTCAACCCCCCATATCTAAAGGTTGACCTCGGATCAGGTAG

>135b3fbbd1c18e2e965fa4da6114c94c

GGGGGCATGCCTGTTCGAGCGTCATTACACCACTCAAGCTATGCTTGGTATTGGGCGTCGTCCTTAGTTGGGCGCGCCTTAAAGACCTCGGCGAGGCCTCACCGGCTTTAGGCGTAGTAGAATTTATTCGAACGTCTGTCAATGGAGAGGACTTCTGCCGACTGAAACCTTTATTTTTTACAGGTTGACCTCGGAT >f7cb27972748505b069e674bde327f5a

TCGGTATTCCGGGGGGCATGCCTGTTCGAGCGTCATTACACCACTCAAGCCTCGGCTTGGTCTTGGGCGTCGCGGTCCCCCGCGTGCCTCAATGTCGCCGGCTGGTGCGACCGTCTCTAAGCGTTGTGAATCAACAGTCGCTCCAGAGGTCGGTCGGGCCCCGCCGTCAAGCCTTTTTTTACAGGTTGACCTCGGATCAGGTAGGGAT

> 26d6fd67c95a1c6e09a748989a8b7994

CAAACCCTCTATCCAACTTTTGTTGAATAGGATGACTGAGAGTCTCTTGATCTATTTTGATCTTGAACCTCTTGAAATGTACAAAGGCCTGATCTTGTTTGAATGCCTGAACTTTTTTTTAATATAAAGAGAAGCTCTTGCGATAAAACTGTGCTGGGGCCTCCCAAATAACACATCTTTAA

> ff6c3a0a4c066e2fc01857c062ee4a10

CTAGTATTCTGGTGGGCATGCCTGTTCGAGCGTCATTTCAACCCTCAAGCCTGGCTTGGTGTTGGGGACCTGCGCACTGCAGTCCCTTAAATCCAGTGGCGGACACGCTAGGTCTCCGAGCGCAGTAGTTTCTTCTCGCTTTGGGCGTCCGGCGTGGGCTTGCCTCGCACCCAACTTCTCAAGGTTGACCTCGGATCAGGTAGGAAT

> 2cbbe38ad49d8c4bad62ca7a1cf3fff8

ATGGGGCATGCCTGTTCGAGCGTCATTTGTACCTTCAAGCCTTGCTTGGTGTTGGGTGTTTGTCTCGCCTCTGCGCGCAGACTCGCCTCAAAACAATTGGCAGCCGGCGTATTGATTTCGGAGCGCAGTACATCTCGCGCTTTGCACTCATAACGACGACGTCCAAAAAGTACATTTTTACACTCTTGACCTCGGATC

> 90d76d3094f07b3c49716abfcfb8492b

GGCATGCCTGTTCGAGCGTCATTTGTACCCTCAAGCTTTGCTTGGTGTTGGGCGTCTTGTCTCTAGCTTTGCTGGAGACTCGCCTTAAAGTAATTGGCAGCCGGCCTACTGGTTTCGGAGCGCAGCACAAGTCGCACTCTCTATCAGCAAAGGTCTAGCATCCATTAAGCCTTTTTTTCAACTTTTGACCTCG

>1fcd05d0b4c69c39e65d5afded0da074

AACCCTCAAACCCAAGTTTTGGATTTCGGTCCATGCTTGAGTTTGGATTTGGATGTTTGCCGGTGATGAACCGACTCATCTTAAAAGTATTAGCTGGATCTGTCTATATGACTGGTTTGACTTGGCATAATAAGTATTTTGCTGAGGACATCTTCGGATGGCCAGGACCTAGACTATTGTCTGCTAACTAAACCATCACTTTAAGTGCATCCTTGGATGTTACTCATTGTGTAACTTTGAC

>5d3e1864ef52b7bbd7d141f0b95a163e

TATTCTGGCGGGCATGCCTGTTCGAGCGTCATTTCAACCCTCAAGCCCAGCTTGGTGTTGGGATCTGTGTGCAAACACAGTCCCCAAATTGATTGGCGGTCACGTCGAGCTTCCATAGCGTAGTAATTTACACATCGTTACTGGTAATCGTCGCGGCCACGCCGTTAAACCCCAACTTCTGAATGTTGACCTCGGATCAGGTAG

>0eb0f9055b30f29822eac6372ced3f14

GCCTGTTCGAGCGTCATTTCAACCCTCAAGCCCCCGGGTTTGGTGTTGGGGATCGGCTCTGCCTTCTGGCGGTGCCGCCCCCGAAATACATTGGCGGTCTCGCTGCAGCCTCCATTGCGTAGTAGCTAACACCTCGCAACTGGAACGCGGCGCGGCCATGCCGTAAAACCCCAACTTCTGAATGTTGA

>43925bad576e342736e3e1b5223b7639

ATGCGATAAGTAGTGTGAATTGCAGAATTCAGTGAATCATCGAATCTTTGAACGCACATTGCGCCCTTTGGTATTCCAAAGGGCATGCCTGTTCGAGCGTCATTTGTACCCTCAAGCTTTGCTTGGTGTTGGGCGTCTTGTCTCTAGCTTTGCTGGAGACTCGCCTTAAAGTAATTGGCAGCCGGCCTACTGGTTTCGGAGCGCAGCACAAGTCGCACTCTCTATCAGCAAAGGTCTAGCATCCATTAAGCCTTTTTTTCAACTTTTGACCTCGGATCAGGTAGGGATACCCGCTGAACTTAAGCATATCAATAAGCGGAGGAAAAGAAACCAACAGGGATTGCCCTAGTAACGG

>47cc5099628737a55d3fa82891b024c6CGTGGGGCATGCCTGTTCGAGCGTCATTTACACCCTCAAGCTCTGCTTGGTGTTGGGCGTCTGTCCCGCCTCCGTGCGCGGACTCGCCTCAAAGTCATTGGCAGCGGTCTCGTCGGCTTCTCGCGCAGCACATTTGCGCTTCTTGGAGCCCCGGCGGATCAGCGTCCAGCAAGCAATTTCATGACTTGACCTCGGATCA

>979f9402723408c308f8a9f9512a2076

ATTCTCCACCCCCATGTCTTTTGTCACCAAAGGACGTGCTTGGGGCGGGTCCATGGGCATCTTTGCCAGTCTTCGGACAGGCTTGCCTTAAAAGTATTAGCTGGACTGCATCTGCGATGCATTGGTTCTACTCAACGTGATAAGTTCATTCGTTGAGGACGGCTTCACGGCCGGCCAAGCTACAATGCGATCAATAGTCTGCTTCTAACCCGGCGCGGAGTGCCTGCACTCCAACCCAACTTAACG

>cfe812b84204fea0c9c3250cae16adaa

TATTCCATGGGGCATGCCTGTTCGAGCGTCATTTGTACCCTCAAGCTCTGCTTGGTGTTGGGTGTTTGTCCACTATCGTGGACTCGCCTTAAAGTCATTGGCAGCCAGTGTTTTGGTATTGAAGCGCAGCACATTTTGCGCCTCTAGCCTAGAACACTAGCGTCCAGTAAGCCTTTTTCCACTTTTGACCTCGGATCAGGTAG

>e6bd9e772840393f1c51a4273613492d

CCGTTACTAAGGCAATCCCTGTTGGTTTCTTTTCCTCCGCTTATTGATATGCTTAAGTTCAGCGGGTATCCCTACCTGATCCGAGGTCAACCTTGAGAAAAGTTCAGAAGGTTCGTCCGGCGGGCGACGCCCTGCGCTCCGAAGCGAGATGTATGTTCTACTACGCTTGAGGCAAGACGCCACCGCCGAGGTCTTTGAGGCGCGCCCGCAAAGGACGGTGCCCAATACCAAGCAGAGCTTGAGGGTTGTAATGACGCTCGAACAGGCATGCCCTTCGGAATACCAAAGGGCGCAATGTGCGTTCAAAGATTCGATGATTCACTGAATTCTGCAATTCACATTACTTATCGCAT

>5a9fbbed7556f8abcc8ff49786274edd

ATGCGATAAGTAATGTGAATTGCAGAATTCAGTGAATCATCGAATCTTTGAACGCACATTGCGCCCCCTGGTATTCCGGGGGGCATGCCTGTTCGAGCGTCATTTCACCACTCAAGCCTCGCTTGGTATTGGGCAACGCGGTCCGCCGCGTGCCTCAAATCGACCGGCTGGGTCTTCTGTCCCCTAAGCGTTGTGGAAACTATTCGCTAAAGGGTGTTCGGGAGGCTACGCCGTAAAACAACCCCATTTCTAAGGTTGACCTCGGATCAGGTAGGGATACCCGCTGAACTTAAGCATATCAATAAGCGGAGGAAAAGAAACCAACAGGGATTGCTCTAGTAACGG

>9eb4329cca14c07f665a91a16ce33d7c

GTATTCCGGGGGGCATGCCTGTTCGAGCGTCATTTCACCACTCAAGCCTCGCTTGGTATTGGGCAACGCGGTCCGCCGCGTGCCTCAAATCGACCGGCTGGGTCTTCTGTCCCCTAAGCGTTGTGGAAACTATTCGCTAAAGGGTGTTCGGGAGGCTACGCCGTAAAACAACCCCATTTCTAAGGTTGACCTCGGATCAGGTAGG

>fb0dcf9010bb8ea5de82c5ccf22547cf

ACCTCTCAAACCCAAGTTTTGGATTTATCCTTGCTTGAGTTTGGATTTGGGTGTTTGCCAGTGATGAACTGACTCACCTTAAAAGTATTAGCTAGATCTGTCTTTGACTGGTTTGACTTGGCATAATAAGTATTTTGCTAAGGACATCTTCGGATGGCCAGGACTTGACTTTTGTCTGCTTACTAAACCTTACTTTAAGTGCATCTCTGGTGTTACTTATAGTATTACT

>c7e12e845a745ca8c6d4c85a3122b5a2

ATGCGATAAGTAATGTGAATTGCAGAATTCAGTGAATCATCGAATCTTTGAACGCACCTTGCGCTCCTTGGTATTCCGAGGAGCATGCCTGTTTGAGTGTCATAAACCTCTCAAACCCAAGTTTTGGATTTATCCTTGCTTGAGTTTGGATTTGGGTGTTTGCCAGTGATGAACTGACTCACCTTAAAAGTATTAGCTAGATCTGTCTTTGACTGGTTTGACTTGGCATAATAAGTATTTTGCTAAGGACATCTTCGGATGGCCAGGACTTGACTTTTGTCTGCTTACTAAACCTTACTTTAAGTGCATCTCTGGTGTTACTTATAGTATTACTTTGACATATGGCCTCAAATCAGGTAGGACTACCCGCTGAACTTAAGCATATCAATAAGCGGAGGAAAAGAAACTAACAAGGATTCCCCTAGTAACGG

>47e640c364f12d4913fbef2ae2742385

CCCCCTGGTATTCCGGGGGGCATGCCTGTTCGAGCGTCATTTCACCACTCAAGCCTCGCTTGGTATTGGGCGTCGCGAGTCTCTCGCGCGCCTTAAAGTCTCCGGCTGTTTGACCCGTCTCCCAGCGTTGTGACAAATTTCGCAGTGGAGTTCGAGTTAACATGGCCGTTAAATCTTTCAAAGGTTGACCTCGGATCAGGTAGGGATACCC

>eb5eb0c8fbb80e2124cf32e45ac986af

AATCTTCAACCCACCTCTTTCTTAGTGAATCTGGTGGTGCTTGGTTTCTGAGCGCTGCTCTGCTTCGGCTTAGCTCGTTCGTAATGCATTAGCATCCGCAACCGAACTTCGGATTGACTTGGCGTAATAGACTATTCGCTGAGGATTCTAGTTTACTAGAGCCGAGTTGGGTTAAAGGAAGCTCCTAATCCTAAAGTCTATTTTTTG
